# Distribution of the gap junction protein connexin 35 in the central nervous system of developing zebrafish larvae

**DOI:** 10.3389/fncir.2013.00091

**Published:** 2013-05-14

**Authors:** Shaista Jabeen, Vatsala Thirumalai

**Affiliations:** National Centre for Biological Sciences, NCBS-TIFRBangalore, India

**Keywords:** whole mount immunohistochemistry, confocal microscopy, dye-coupling, optic tectum, electrical synapse, electrotonic coupling, cerebellum

## Abstract

Gap junctions are membrane specializations that allow the passage of ions and small molecules from one cell to another. In vertebrates, connexins are the protein subunits that assemble to form gap junctional plaques. Connexin-35 (Cx35) is the fish ortholog of mammalian Cx36, which is enriched in the retina and the brain and has been shown to form neuronal gap junctions. As a first step toward understanding the role of neuronal gap junctions in central nervous system (CNS) development, we describe here the distribution of Cx35 in the CNS during zebrafish development. Cx35 expression is first seen at 1 day post fertilization (dpf) along cell boundaries throughout the nervous system. At 2 dpf, Cx35 immunoreactivity appears in commissures and fiber tracts throughout the CNS and along the edges of the tectal neuropil. In the rhombencephalon, the Mauthner neurons and fiber tracts show strong Cx35 immunoreactivity. As the larva develops, the commissures and fiber tracts continue to be immunoreactive for Cx35. In addition, the area of the tectal neuropil stained increases vastly and tectal commissures are visible. Furthermore, at 4–5 dpf, Cx35 is seen in the habenulae, cerebellum and in radial glia lining the rhombencephalic ventricle. This pattern of Cx35 immunoreactivity is stable at least until 15 dpf. To test whether the Cx35 immunoreactivity seen corresponds to functional gap junctional coupling, we documented the number of dye-coupled neurons in the hindbrain. We found several dye-coupled neurons within the reticulospinal network indicating functional gap junctional connectivity in the developing zebrafish brain.

## Introduction

Ever since electrical synapses were first described (Furshpan and Potter, 1957), their role in the formation and function of neural circuits has been the focus of intense study. Electrical synapses or neuronal gap junctions are formed by associations of protein subunits that form a functional hemi-channel on each partner neuron membrane. The coming together of two hemi-channels forms a continuous pore between the partner neurons through which ions and small molecules can be exchanged (Söhl et al., 2005). Gap junctions are formed by innexin subunits in invertebrates and connexin subunits in vertebrates. Connexin and innexin protein families are unrelated, while the vertebrate homolog of innexins, the pannexins, are thought to be not involved in gap junction assembly (Sosinsky et al., 2011; Abascal and Zardoya, 2013).

Gap junctions are present on neurons as well as in glia, vascular smooth muscle, inner ear hair cells, pancreatic epithelial cells and in multiple cell types in the retina (Kar et al., 2012). However, gap junctions in these diverse tissues are assembled from distinct connexin subunits. Twenty connexin genes have been identified in the mouse genome, 21 in the human and the same number of innexin genes is present in the medicinal leech, *Hirudo verbana* (Kandarian et al., 2012). Connexin genes and their protein products are named by the molecular weight of the protein—Cx43 stands for a connexin subunit of 43 kDa. Among these connexin family proteins, (Connexin-35) Cx35/Cx36, Cx40, Cx45, and Cx57 have been shown to form neuronal gap junctions (Söhl et al., 2005). Cx36 is widely expressed in the central nervous system (CNS) of mammals, notably in the dendrites of inferior olivary neurons, and in interneurons of the hippocampus, cerebellum, and cerebral cortex (Condorelli et al., 2000; Söhl et al., 2005). Although the functional significance of Cx36-mediated electrical synapses in the CNS is not yet completely understood, there is some evidence that it may be involved in increasing synchrony among oscillating neurons (Connors and Long, 2004). During development, neuronal gap junctions are transiently increased ahead of chemical synaptogenesis. This has led to the notion that gap junctions may prefigure chemical synapses, an idea that has gained support from gap junction perturbation experiments in the leech (Todd et al., 2010). However, mice lacking Cx36 [Cx36^(−/−)^] did not exhibit major deficits in physiology or behavior (Connors and Long, 2004; Söhl et al., 2005). Because null mutants may compensate for the loss of one isoform of connexin with another, or use alternate mechanisms of development, the function of Cx35/36 during development needs to be better-addressed using techniques that allow greater spatial and temporal specificity in knocking down gene expression. Toward this end, we have started to study gap junctions in the small tropical teleost, *Danio rerio*, where such experiments are elegantly possible.

Although there are four orthologs in zebrafish for the mammalian Cx36, Cx35 is the only ortholog that shows high similarity (~90%) to Cx36 and has been shown to be expressed and enriched in the CNS. It has been previously demonstrated to be present in gap junctions formed by the Mauthner neuron in goldfish and zebrafish (Pereda et al., 2003; Satou et al., 2009). Cx35 is present on mixed synapses formed by auditory nerve afferents on the Mauthner neuron lateral dendrite where Cx35 is in close association with NMDA receptors (Rash et al., 2004). Cx35 is a 962 base pair gene with two exons located on chromosome 20 and encodes a 304 amino-acid protein. The zebrafish Cx35 shares a 99% similarity with perch Cx35 and a 90% similarity with mouse and human Cx36. Cx35 protein was detected in punctate form in the photoreceptors and the inner plexiform layer of the retina in larval zebrafish (McLachlan et al., 2003; Li et al., 2009). However, the distribution and ontogeny of Cx35 within the CNS of zebrafish have not yet been studied. Here, using whole-mount immunohistochemistry, we describe the distribution of Cx35-like immunoreactivity in zebrafish larvae from 1 day post fertilization (dpf) to 15 dpf in different regions of the brain. We also show that neurons in the hindbrain of larval zebrafish are extensively dye-coupled, suggesting that the Cx35 immunoreactive puncta that we observe assemble into functional gap junctions.

## Materials and methods

Adult wild type zebrafish *(Danio rerio)* were obtained from a commercial supplier, housed in a recirculating water system (Tecniplast, Italy) and used for generating embryos. All procedures were approved by the Institutional Animal Ethics Committee, National Centre for Biological Sciences.

### Whole-mount immunohistochemistry

Zebrafish larvae were staged based on external morphological features (Kimmel et al., 1995). Larvae from 1 to 6 dpf, 10 and 15 dpf were anaesthetized in ethyl 3-aminobenzoate methane sulfonate (MS-222; 0.01% w/v) and then were fixed in 4% para-formaldehyde (PFA) for 12–14 h at 4°C. Fixed larvae were washed with 0.1 M phosphate-buffered saline (PBS). Larvae were pinned down using 0.001” diameter Tungsten wire (California Fine Wire Company, Grover Beach, CA) on a petri dish lined with Sylgard (Dow Corning, Midland, MI). Skin from the top of the head was gently peeled off and the yolk sac, eyes, jaws, and palate were removed carefully leaving only the brain and the tail region containing the spinal cord. Dissected larvae were then blocked overnight in 3 mg/ml normal donkey serum (Jackson Immuno Research, West Grove, PA) in 0.1 M PBS and 0.5% Triton X-100 (PBST) followed by incubation for 48 h at 4°C with mouse anti-Cx35 antibody (MAB3045, EMD Millipore, Billerica, MA) at 1:200 dilution. After washing larvae in PBST several times, they were incubated with donkey anti-mouse IgG-coupled with Dylight 649 (Jackson Immuno Research) or Alexa Fluor 488 (Invitrogen, Whitefield, Bangalore) at 1:500 dilution for 12 h at 4°C. Larvae were then washed several times with cold 0.1 M PBS and mounted with Prolong Gold Anti-Fade reagent (Invitrogen) between two cover slips for imaging. Stacks of images were taken using a Zeiss LSM 510 meta NLO confocal microscope under optimal intensity conditions. Images were taken using 40X oil immersion objective with z-spacing set at 0.44 μm. The numbers of animals used in each stage is given in Table 1.

**Table 1:** **Numbers of larvae imaged at each stage**.

**Age**	**1 dpf**	**2 dpf**	**3 dpf**	**4 dpf**	**5 dpf**	**6 dpf**	**10 dpf**	**15 dpf**
Animals (n)	5	7	5	5	11	11	3	7

### Retrograde labeling

Larvae were anaesthetized in ethyl 3-amino benzoate methane sulfonate (MS-222; 0.01% w/v). A mixture of 25% w/v Neurobiotin (Vector Laboratories; 287Da) and 25% w/v tetra methyl rhodamine-dextran (Invitrogen; 3 kDa), dissolved in autoclaved, filtered and deionized water was pressure injected into the spinal cord of the anaesthetized larvae using a Pico-spritzer III (Parker Hannifin Corp, Pine Brook, NJ). In some larvae, only tetra-methyl rhodamine dextran was injected and these larvae were subsequently processed for whole-mount immunostaining as described above. Larvae were allowed to recover for 24 h in Hank's solution (137 mM NaCl, 5.4 mM KCl, 0.25 mM Na_2_HPO_4_, 0.44 mM KH_2_PO_4_, 1.3 mmCaCl_2_, 1.0 mM MgSO_4_, 4.2 mM NaHCO_3_). After recovery larvae were examined under the Olympus SZX16 epifluorescence stereo-microscope for dye labeling in the hindbrain. Larvae having dye-filled neurons were euthanized in cold MS-222 followed by fixation in 4% w/v PFA for 12–14 h. Brain and spinal cord were then dissected out as described above and processed further with 5μg/ml Streptavidin-Alexafluor-488. Larvae were then imaged under a Zeiss LSM 510 meta NLO confocal microscope using the appropriate excitation wavelengths and filters.

### Figure production and analysis

Images were viewed offline using Fiji (http://fiji.sc/) and figures were produced using Adobe Photoshop. The neurobiotin-filled cells were counted using plug-ins in Fiji and results were tabulated in Microsoft Excel.

## Results

We used a monoclonal antibody raised against perch Cx35 to study the distribution of Cx35 in whole-mount larval zebrafish from 1 to 15 dpf. This antibody has been previously shown to bind specifically to perch and zebrafish Cx35. Western blots on brain lysates showed no cross-reactivity with other connexin isoforms (Pereda et al., 2003; Satou et al., 2009). Therefore, we proceeded to perform whole-mount immunohistochemistry using this antibody and found reliable staining from identifiable anatomical structures. The Cx35 immunoreactivity was punctate and was seen in both neuropil and membranes of cell bodies, as would be expected of Cx35. Further, no staining was seen when the primary or the secondary antibodies were omitted from the incubation mixture. In all preparations except in 1 dpf larvae, the eye was dissected out and therefore we do not discuss the retinal staining pattern, which can be found elsewhere (McLachlan et al., 2003; Li et al., 2009). Here we describe the development of Cx35 immunoreactivity in the CNS of zebrafish from 1 dpf through 15 dpf.

### 1 dpf

**Figure 1:**
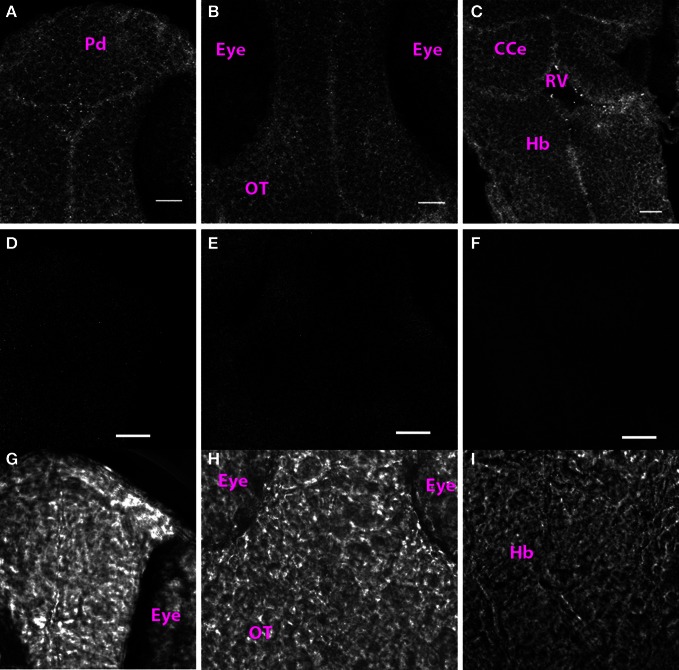
**Cx35 staining pattern in 1 dpf larva. (A)** Single optical section of the dorsal pallium (Pd). **(B)** Optic tectum (OT). **(C)** Hindbrain (Hb). The cerebellum (CCe), and the rhombencephalic ventricle (RV) can be also be seen. Anterior is at the top and posterior is at the bottom. Larva is in dorsoventral position. **(D–F)** Single optical sections from control larvae in which the primary antibody was not included in the incubation mixture. **(D)** Dorsal telencephalon. **(E)** Optic tectum. **(F)** Hindbrain. **(G–I)** Transmitted light images of the regions imaged in **(D–F)**. Scale bar for all panels: 20 μm.

At 1 dpf, we found punctate Cx35 labeling along cell membranes throughout the rostrocaudal extent of the CNS (Figures 1A–C). This staining was specific because omission of the primary antibody from the incubation mixture abolished all signal (Figures 1D–F: fluorescence image; Figures 1G–I show the respective transmitted light images). No defined structures such as nuclei, commissures or tracts were visible at this stage.

### 2 dpf

**Figure 2:**
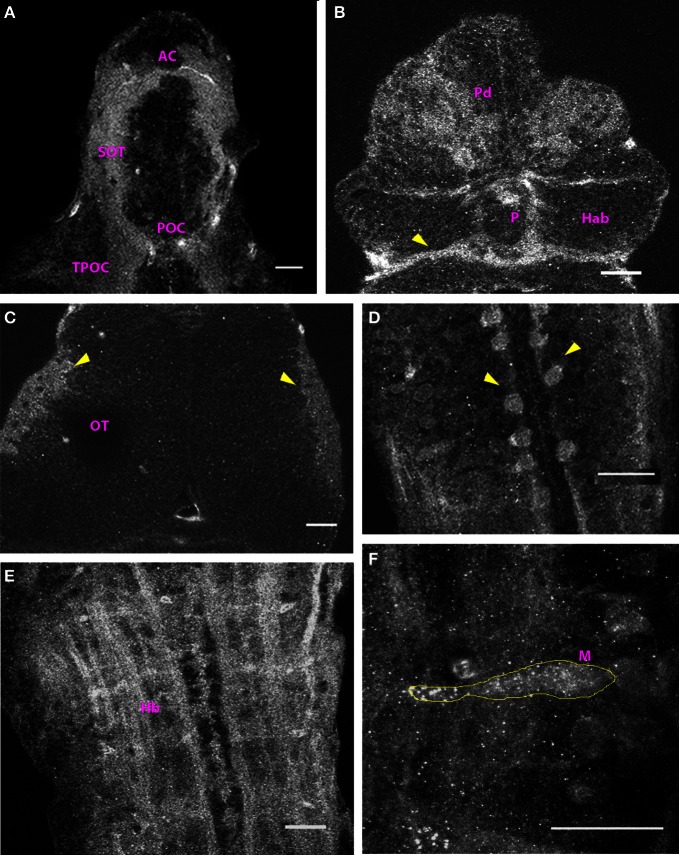
**Cx35 distribution in the CNS of 2 dpf larvae.** Larvae are dorso-ventrally placed with anterior on top in all panels. All panels except **(F)** show single optical sections taken at a z-resolution of 0.44 μm. **(A)** Ventral telencephalon showing the anterior commissure (AC), supra-optic tract (SOT), and the post-optic commissure (POC). **(B)** Dorsal view of forebrain showing Cx35 positive cells in the dorsal pallium (Pd). The posterior commissure can be seen (arrowhead) while the habenulae (Hab) and the pineal body (P) lack Cx35 immunoreactivity. **(C)** Dorsal view of the optic tectum (OT). A single optical section showing Cx35 immunoreactivity in the developing neuropil region (arrowheads). **(D)** Cell bodies (arrowheads) in hindbrain lining the rhombencephalic ventricle. **(E)** Ventral hindbrain (Hb) showing intense Cx35 immunoreactivity in fibers coursing through it. **(F)** Zoomed image of a Mauthner neuron (M, yellow outline) showing punctate Cx35 immunoreactivity. The image is a maximum intensity projection of optical slices collected from a 16 μm thick tissue slice encompassing the Mauthner neuron. Scale bar for all panels: 20 μm.

At 2 dpf, the anterior and the post-optic commissures, the tract of the post optic commissure and the supra-optic tract were labeled in the ventral forebrain (Figure 2A). Cx35-positive ellipsoid cells were found in the dorsal pallium (Figure 2B). Thick fiber tracts were present in the ventral diencephalon and the posterior commissure was prominently labeled in the dorsal aspect (Figure 2B, arrowhead). The rim of the optic tectal neuropil was Cx35 positive but no other fiber tracts or cells were labeled in the mesencephalon (Figure 2C). In the rhombencephalon, large rounded cells were seen lining the rhombencephalic ventricle. No processes were visible on these cells (Figure 2D). The hindbrain displayed intense staining in the ventral aspect, with fiber tracts labeling strongly with the anti-Cx35 antibody. Prominently, fiber tracts in the lateral longitudinal fascicle were labeled and a ladder-like pattern was visible (Figure 2E). A little more dorsally, the Mauthner neurons were clearly labeled with the antibody (Figure 2F). Many other cells were also faintly stained in the vicinity of the Mauthner neurons.

### 3 dpf

**Figure 3:**
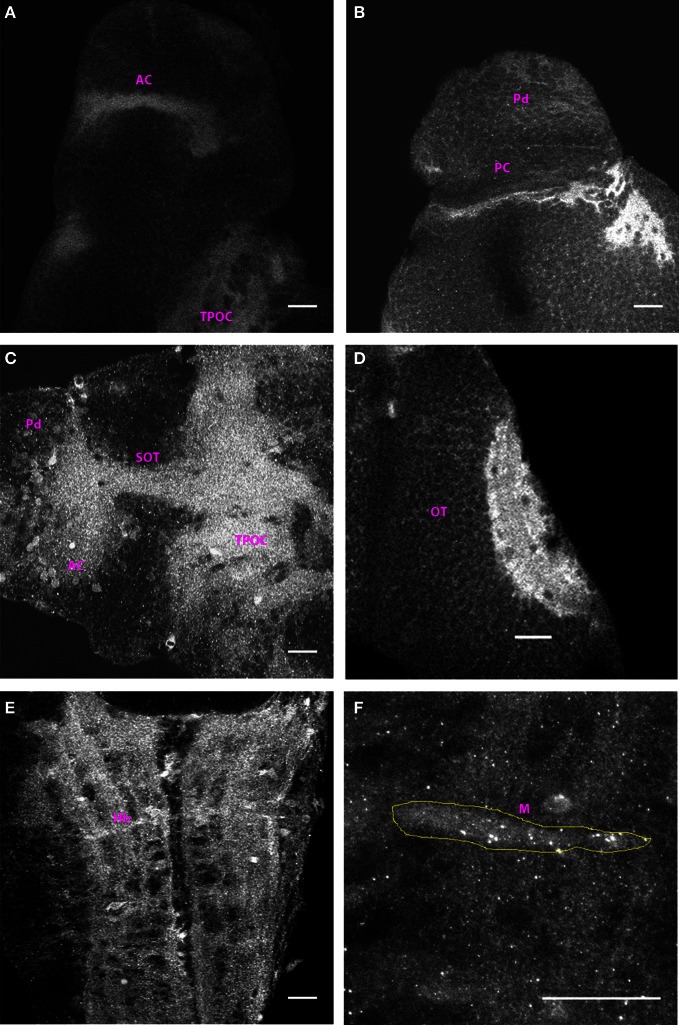
**Distribution of Cx35 immunoreactivity in 3 dpf larvae.** All panels except **(C)** show dorso-ventrally placed larvae, with the anterior end of the animal at the top of the panel. All panels are single optical sections taken at a z-resolution of 0.44 μm. **(A)** Ventral forebrain showing immunoreactivity in the anterior commissure (AC) and in the tract of the post-optic commissure (TPOC). **(B)** Dorsal telencephalon and rostral mesencephalon. Staining in cells of the dorsal pallium (Pd) and in the posterior commissure (PC) can be seen. **(C)** Lateral view of forebrain. Dorsal is on top and anterior to the left. Cx35 immunoreactivity is seen in the anterior commissure, in the supra-optic tract and in thick fiber tracts in ventral diencephalon. Numerous cell bodies can also be seen in the vicinity of the anterior commissure. **(D)** Intense Cx35 immunoreactivity in the optic tectal (OT) neuropil. **(E)** Ventral hindbrain (Hb) showing fiber tracts criss-crossing the hindbrain. **(F)** Zoomed image of a single Mauthner neuron (M) showing intense Cx35 immunoreactive puncta. The approximate boundary of the Mauthner neuron as seen in DIC is shown in yellow. Scale bar for all panels: 20 μm.

The Cx35 staining pattern at 3 dpf was very largely similar to that seen at 2 dpf. As at 2 dpf, the anterior and post-optic commissures along with the tract of the post-optic commissure were labeled (Figures 3A and C). Dorsally, cells in the pallium were intensely labeled as was the posterior commissure (Figure 3B). The tectal neuropil area stained was larger as would be expected with the ingrowth of retinal ganglion cell axons and tectal axons into this region. No other fiber tracts or cells were stained in the mesencephalon at this stage (Figure 3D). In the rhombencephalon, the ladder-like pattern of fiber tracts (Figure 3E), the Mauthner neurons and several hindbrain cell bodies in its vicinity could be seen labeled. The Mauthner neuron showed sharp intense puncta of Cx35 staining across its cell body (Figure 3F). However, we failed to observe any cells lining the rhombencephalic ventricle at this stage.

### 4 dpf–15 dpf

**Figure 4:**
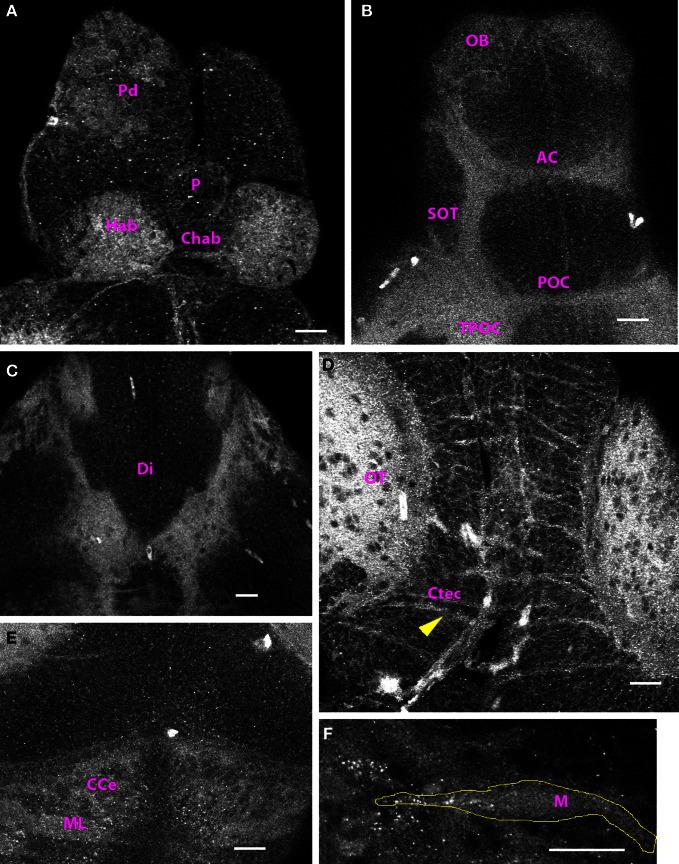
**Cx35 immunostaining in 4 dpf larvae.** Larvae are dorso-ventrally placed with anterior on top in all panels. All panels show single optical sections taken at a z-resolution of 0.44 μm. **(A)** Dorsal forebrain showing Cx35 staining in the paired habenulae (Hab) and in the habenular commissure (Chab). Cell bodies in dorsal pallium (Pd) continue to stain positive. No staining is seen in the pineal body (P). **(B)** Ventral telencephalon showing Cx35 immunoreactivity in the olfactory bulb (OB), anterior commissure (AC), supra-optic tract (SOT), post-optic commissure (POC), and the tract of the post-optic commissure (TPOC). **(C)** Cx35 immunoreactivity in fiber tracts in the ventral diencephalon (Di). **(D)** Intense Cx35 immunoreactivity in the optic tectal neuropil (OT) and in the intertectal commissures (Ctec; arrowhead) in the mesencephalon. Tectal cell bodies are also weakly labeled. **(E)** Weak staining is seen in the cerebellum within the molecular layer (ML) and in the corpus cerebelli (CCe). **(F)** Zoomed image of a single Mauthner neuron (M) showing intense Cx35 immunoreactive puncta. The approximate boundary of the Mauthner neuron as seen in DIC is shown in yellow. Scale bar for all panels: 20 μm.

By 4 dpf, the distribution of Cx35-immunoreactivity was almost fully mature and the structures labeled at 4 dpf continued to be Cx35-positive at least until 15 dpf, the oldest stage examined by us. In the telencephalon, cell bodies in the dorsal pallium could be clearly seen (Figure 4A). In addition, at 4 dpf, the habenulae were brightly labeled and the habenular commissure was also visible (Figure 4A). Ventrally, the supra-optic tract and the anterior and post-optic commissures continued to be labeled strongly (Figure 4B). In addition, cell bodies in the olfactory bulb showed intense labeling. Staining was also seen in the glomerular region (Figure 4B). Ventrally, thick fiber tracts continuing from the tract of the post-optic commissure could be seen in the diencephalon (Figure 4C). In the mesencephalon, the neuropil of the tectum was the most brightly-labeled structure in the entire CNS (Figure 4D). Puncta dotted the outlines of cell bodies in the tectal lobes and tectal commissures were seen to run across the lobes. Paired longitudinal tracts were also seen medially in dorsal tectum. Beginning at 4 dpf, Cx35 immunoreactivity could be seen in the cerebellum. The molecular layer was prominent and staining was seen in the climbing fibers as well as in the corpus cerebelli (Figure 4E). Similarly, the hindbrain was also brightly stained with the ladder-like fiber tracts. Intense puncta were visible on the Mauthner neuron somata (Figure 4F).

**Figure 5:**
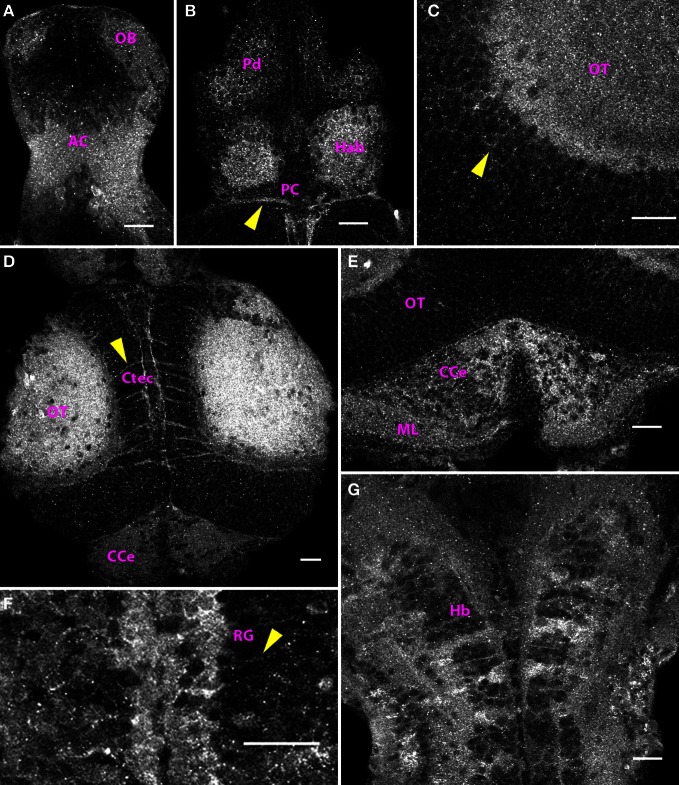
**Distribution of Cx35 immunoreactivity in the CNS of 6 dpf larvae.** Larvae are dorso-ventrally placed with anterior on top in all panels. All panels show single optical sections taken at a z-resolution of 0.44 μm. **(A)** Ventral telencephalon showing staining in anterior commissure (AC) and the olfactory bulb (OB). A few faintly stained cell bodies can be seen in the vicinity of the anterior commissure. **(B)** Dorsal forebrain showing Cx35 staining in the habenulae (Hab), dorsal pallium (Pd), and the posterior commissure (PC; arrowhead). **(C)** Magnified dorsal view of the optic tectum (OT) showing intense staining in the neuropil area. Faint labeling in cell bodies is also seen (arrowhead). **(D)** Cx35 staining in the mesencephalon showing intense staining in the optic tectal lobes in the neuropil area as well as in intertectal commissures (Ctec; arrowhead). The corpus cerebelli (CCe) can also be seen. **(E)** Cerebellar staining showing punctate pattern within the corpus cerebelli (CCe) and the molecular layer (ML). **(F)** Strong staining present in radial glia (RG) lining the rhombencephalic ventricle. Arrowhead marks the process from one glial cell coursing laterally. **(G)** Ladder-like staining pattern in ventral hindbrain (Hb). Scale bar for all panels: 20 μm.

Subsequently, at 5 dpf and 6 dpf, the distribution of Cx35 immunoreactivity was similar to what was seen at 4 dpf (Figure 5). In ventral telencephalon, the anterior commissure and the olfactory bulb were labeled (Figure 5A). In dorsal telencephalon, cell bodies in the Pallium and the habenulae could be clearly seen. The posterior cimmissure was also labeled (Figure 5B). In the mesencephalon, the much larger tectal neuropil was strongly stained (Figures 5C,D) and the outlines of tectal cell bodies were marked by Cx35-positive puncta (Figure 5C). Intertectal commissures and paired longitudinal tracts running in medial tectum were clearly visible (Figure 5D). As in the 4 dpf larvae, the cerebellum was also strongly Cx35-immunoreactive (Figure 5E). Additionally, beginning at 5 dpf, radial glia lining the rhombencephalic ventricle were seen to express Cx35 and their straight processes were seen to traverse the hindbrain from the medial to the lateral margins (Figure 5F). Strong Cx35 staining was also seen in ventral hindbrain, in a pattern similar to that seen at 4 dpf.

**Figure 6:**
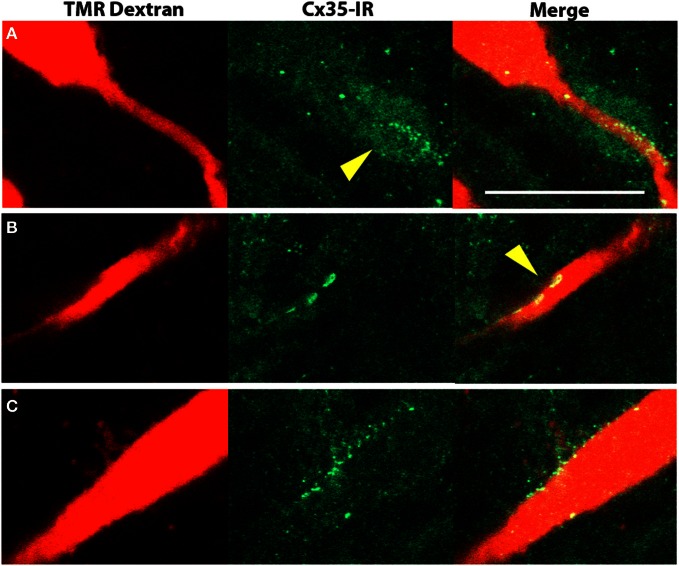
**Cx35 immunoreactivity in Mauthner neurons of 6 dpf larvae.** The left panels show retrograde labeling of a Mauthner neuron with tetra-methyl rhodamine dextran (TMR dextran, red). The middle panels show Cx35 immunoreactivity in green and the right panels show merge of the two channels. All images are single optical sections taken at a z-spacing of 0.44 μm. **(A)** Cx35-immunoreactive puncta line the Mauthner axon and form a dense cloud in the axon-cap region (arrowhead). **(B)** Club endings on the lateral dendrite are large bouton-like structures (arrowhead) that are intensely Cx35 immunoreactive. **(C)** Cx35 puncta are also seen to line the Mauthner soma. Scale bar for all panels: 20 μm.

In 5–6 dpf larvae, we could see bright puncta of Cx35 immunoreactivity in the Mauthner neurons. To study the placement of these puncta on the Mauthner neuron, we retrogradely labeled the Mauthner neuron with tetra methyl rhodamine dextran and then processed the larvae for Cx35 immunoreactivity. We found puncta lining the axon in the axon-cap region of the Mauthner neuron (Figure 6A), on the lateral dendrite at club-endings (Figure 6B) and on the soma (Figure 6C).

**Figure 7:**
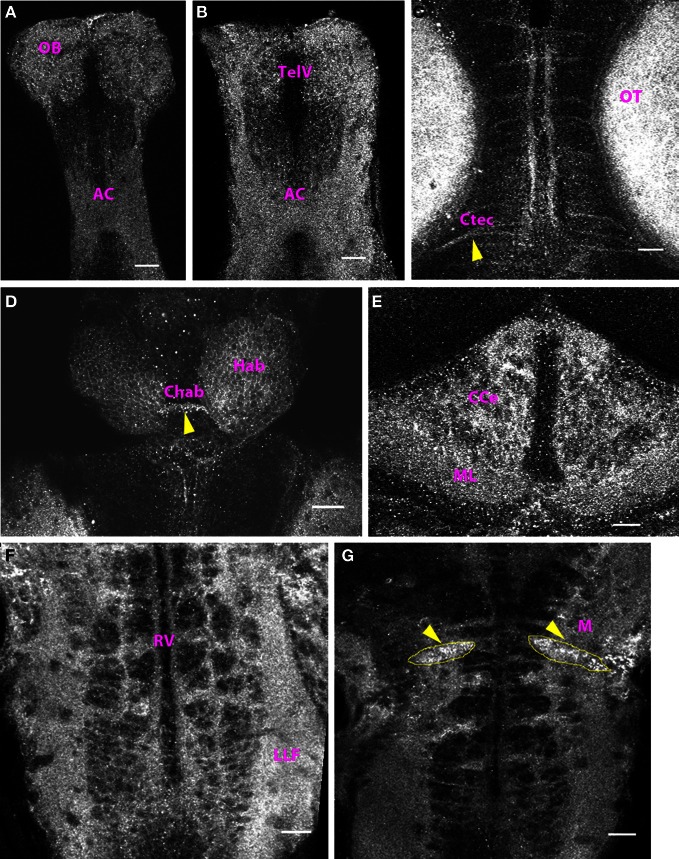
**Cx35 staining pattern in the CNS of 15 dpf zebrafish.** Larvae are dorso-ventrally placed with anterior on top in all panels. All panels show single optical sections taken at a z-resolution of 0.44 μm. **(A)** Ventral telencephalon showing punctate Cx35 immunoreactivity in the anterior commissure (AC) and the olfactory bulb (OB). **(B)** Telencephalon 50 μm from the ventral surface showing staining in the anterior commissure (AC) and in the olfactory bulb (OB). The telencephalic ventricle (TelV) can be seen. **(C)** Mesencephalon, dorsal view showing staining in the optic tectal (OT) neuropil and in intertectal commissures (Ctec, arrowhead). **(D)** Habenulae (Hab) and the habenular commissure (Chab; arrowhead). Polygonal cell bodies can be seen in the habenulae **(E)** Cerebellum showing intense Cx35 immunoreactivity in the corpus cerebelli (CCe) and the molecular layer (ML) **(F)** Ventral Hindbrain (Hb) showing a ladder-like pattern that includes Cx35 staining in the lateral longitudinal fasciculus (LLF) and hindbrain commissures. The rhombencephalic ventricle (RV) can be seen. **(G)** Intense punctate Cx35 immunoreactivity in the Mauthner neurons (M; arrowheads). The approximate boundaries of the Mauthner neurons is drawn in yellow. Scale bar for all panels: 20 μm.

At 10 dpf (data not shown) and 15 dpf, the distribution pattern of Cx35 immunoreactivity was similar to that seen at 6 dpf. The regions that stained positive at 6 dpf, continued to stain positive through 15 dpf and no new regions were seen (Figure 7).

### Dye-coupling in reticulospinal neurons

**Figure 8:**
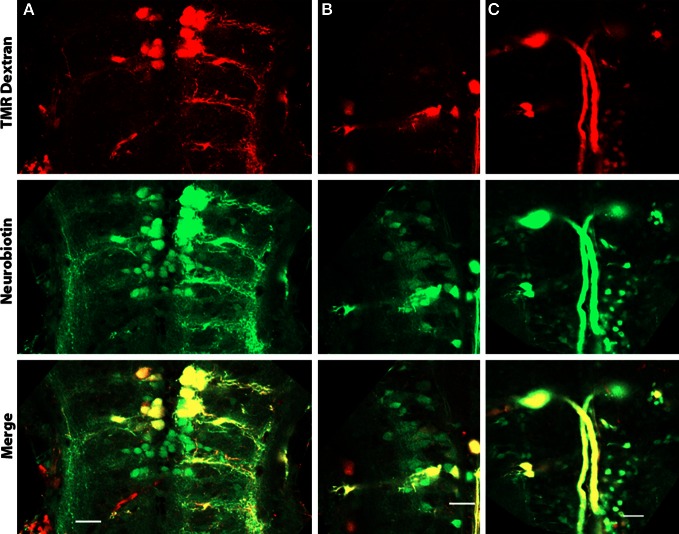
**Dye-coupled cells in the hindbrain of 4 dpf larvae.** Larvae are dorso-ventrally placed with anterior on top in all panels. All panels show single optical sections taken at a z-resolution of 0.44 μm. **(A–C)** Images at depths of 20, 30, and 43 μm from the ventral surface showing reticulospinal neurons retrogradely labeled with neurobiotin (green) and tetramethyl rhodamine dextran (red). Cells electrically coupled to the labeled neurons but not projecting into the spinal cord are green in color, while the neurons that project into the spinal cord are yellow because of the presence of both neurobiotin and tetra methyl rhodamine dextran. Scale bar for all panels: 20 μm.

We sought to determine if the high intensity of Cx35 immunoreactivity in the hindbrain of zebrafish larvae indicates a high level of gap junction connectivity between hindbrain neurons. We retrogradely labeled reticulospinal neurons by pressure injecting a 50:50 mixture of neurobiotin and tetra-methyl rhodamine dextran into the spinal cord. These dyes are retrogradely transported by the spinal axons to their respective somata in the hindbrain. While tetra methyl rhodamine dextran stays within the same cell, neurobiotin can cross any gap junctions due to its small mass (287 Da). When the neurobiotin is visualized using fluorophore-coupled streptavidin, while the cells directly labeled retrogradely colocalize both neurobiotin and the higher molecular weight dextran, the dye-coupled cells are only positive for neurobiotin (Figure 8). We counted the number of neurons labeled with neurobiotin only and normalized it to the number of retrogradely labeled neurons (containing both neurobiotin and tetra-methyl rhodamine dextran). This yields a dye-coupling ratio, which indicates the number of neurons with which a single reticulospinal neuron may share gap junctions. Between 4 dpf and 6 dpf, we found a dye-coupling ratio of 2.5 ± 0.7 (Mean ± SD, *n* = 6), indicating a high degree of gap junctional coupling between reticulospinal neurons and other hindbrain neurons.

## Discussion

**Figure 9:**
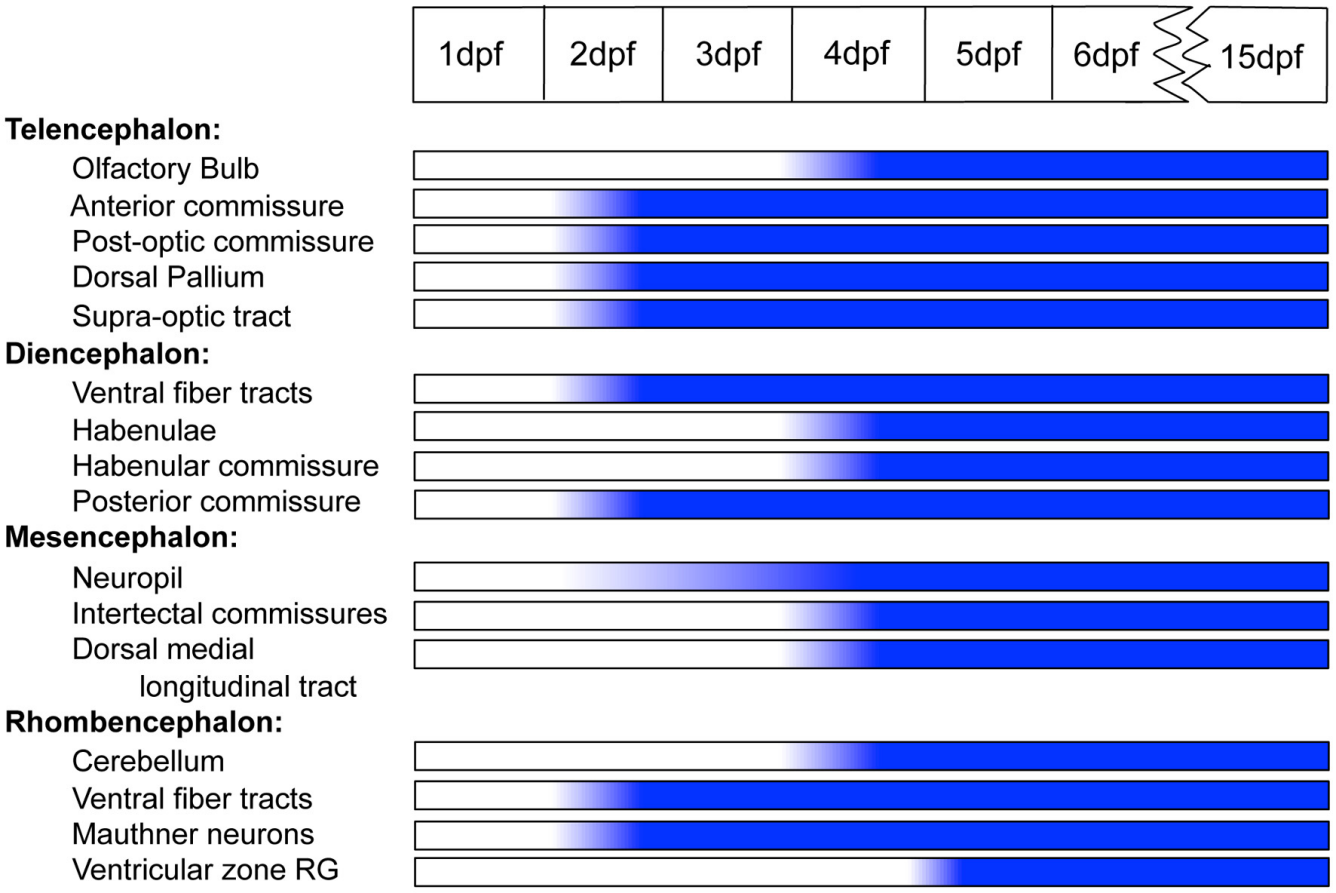
**Timeline showing the ontogeny of Cx35 immunoreactivity in the CNS of zebrafish from 1 dpf through 15 dpf.** Abbreviations are as given in the text.

We have followed the appearance of Cx35 immunoreactivity in the CNS of embryonic and larval zebrafish from 1 dpf through 15 dpf. We find that while some structures such as the Mauthner neurons and the commissures acquire Cx35 immunoreactivity relatively early (2 dpf), other structures such as the habenulae and the cerebellum begin to express Cx35 only by 4 dpf. However, on the whole, the Cx35 distribution pattern seems to mature in the early larval stage and remains stable into late larval stages (Figure 9).

We used a monoclonal antibody raised against perch Cx35, which shows specificity for Cx35 in perch, zebrafish and gold fish (Pereda et al., 2003; Satou et al., 2009). As noted earlier, perch Cx35 and zebrafish Cx35 share a 96% identity and 99% similarity at the amino acid level, therefore it is not surprising that this antibody specifically recognizes the zebrafish Cx35 protein. Earlier this antibody was shown to detect gap junctional contacts between the Mauthner axon and a spinal inhibitory interneuron in zebrafish larvae (Satou et al., 2009). Furthermore, this antibody also specifically detects Cx35-mediated gap junctional plaques in the Mauthner neuron of goldfish (Pereda et al., 2003). There are four known orthologs of mammalian Cx36 in zebrafish—Cx35 on chromosome 20 and three other genes on chromosomes 5, 7, and 17. While there is currently no evidence that the ortholog on chromosome 17 is expressed, the other two isoforms have very low similarity to the intracellular loop of the perch Cx35, the antigen against which the antibody was raised. Furthermore, western blots show no cross-reactivity with other connexin isoforms (Pereda et al., 2003). Therefore, our results indicate true Cx35 distribution within the CNS of developing zebrafish larvae.

We found that Cx35 distribution in the zebrafish CNS recapitulates Cx36 distribution in the mammalian CNS to a large extent. For example, Cx36 is known to be present in the retina, olfactory bulb, cerebellum, habenula, inferior olive and other brain stem nuclei in the adult rodent brain (Condorelli et al., 2000; Söhl et al., 2005). Earlier studies have demonstrated Cx35 presence in the retina of zebrafish between photoreceptors and in the inner plexiform layer (McLachlan et al., 2003; Li et al., 2009). Our studies now show that Cx35 is present in many regions of the CNS including the olfactory bulb, cerebellum, habenulae, and the hindbrain. Together, these results suggest that Cx35 and Cx36 are not only homologous with respect to their sequence but might serve similar functions in homologous areas of the teleost and mammalian brains.

While an earlier study indicated that Cx35 is not present at 1 dpf (McLachlan et al., 2003), our immunostaining results show that a low level of Cx35 is present throughout the rostrocaudal extent of the larval CNS. These results are also supported by recent preliminary experiments from another group (Martin and Ribera, 2012). Beginning at 2 dpf, we observed that the anterior, post-optic and posterior commissures exhibit Cx35 immunoreactivity. Additionally, fiber tracts in ventral diencephalon and in ventral hindbrain were labeled. This suggests gap-junctional contacts between axons, especially since axons are not myelinated at 2 dpf (Brösamle and Halpern, 2002; Jung et al., 2010). The mammalian ortholog of Cx35, Cx36 is indeed found along hippocampal mossy fibers where pairs of mossy fibers were found to be electrically coupled via Cx36 containing plaques (Hamzei-Sichani et al., 2007). Such axo-axonic electrical coupling may serve to spread action potentials from one axon to another and synchronize populations of neurons, resulting in oscillations. We found Cx35 immunoreactivity along many commissures and fiber tracts throughout the rostro-caudal extent of the zebrafish CNS. The functional relevance of such extensive electrical coupling remains to be evaluated.

The optic tectal neuropil showed intense Cx35 immunoreactivity. The extent of staining seen increased with development as the neuropil grew (see Figures 2C, 3D, 4D, 5D, and 7C). We see Cx35 immunoreactivity begin to appear within the growing tectal neuropil as early as 2 dpf, a stage when retinal axons have not yet innervated their target region within the tectum (Stuermer, 1988). This suggests that Cx35-mediated gap junctions might be present in contacts between tectal neurons as they grow their neurites into the tectal neuropil. We also found Cx35 puncta dotting the boundaries of tectal cell bodies (for e.g., Figure 5C) suggesting gap junctional connectivity between somata in the tectum. Physiological validation of such electrical coupling and its function in visual processing remain to be investigated.

In contrast to the optic tectum, Cx35 immunoreactivity in the habenulae and the cerebellum developed later, starting at 4 dpf. Although the habenulae and the habenular commissure can be distinguished by 2 dpf (Hendricks and Jesuthasan, 2007), we did not see Cx35 immunoreactivity in them until 4 dpf. We saw intense labeling in habenular neurons and in the commissure indicating gap junctional connectivity among habenular neurons from 4 dpf onwards. Cerebellar glutamatergic and GABAergic neurons are first detected at 3 dpf and a layered structure is first seen at 5 dpf. By 5 dpf, vGlut1-positive presynaptic terminals contact dendrites from Purkinje neurons and a rapid rate of synapse formation continues at least until 15 dpf (Bae et al., 2009). We find it significant that Cx35 immunoreactivity is seen immediately prior to the start of synaptogenesis in the cerebellum. In many systems, electrical synapses have been shown to be upregulated before the period of chemical synaptogenesis and recently, knocking down gap junctions was shown to interfere with chemical synapse formation (Todd et al., 2010). It is likely that in the cerebellum, Cx35-mediated gap junctions provide instructional cues for the formation of appropriate chemical synaptic connections.

The Mauthner neurons are large neurons in rhombomere 4 that are critical for escape behavior. Electrical synapses on the Mauthner neurons are numerous: they are present in mixed synapses formed by the eighth cranial nerve club endings and in spiral fiber endings on the axon cap. These gap junctions are known to be present by 2 dpf (Kimmel et al., 1981) and the gap junctions at club endings are known to be mediated by Cx35 in goldfish (Pereda et al., 2003). Consistent with these data, we found intense Cx35 immunoreactivity in Mauthner neuron somata beginning at 2 dpf and at least until 15 dpf, suggesting the presence of Cx35-mediated gap junctions. Club endings are known to be located on the lateral dendrite of the Mauthner neuron, whereas spiral fiber endings are on the axon cap (Kimmel et al., 1981). Consistent with this, we see bright and intense puncta in these locations (Figure 6). Notably, we see large button-like puncta whose shape is characteristic of the club endings formed by the eighth cranial nerve (Figure 6B). We also see several dimly stained cell bodies in the hindbrain. Since their morphology could not be traced with the antibody staining or with retrograde labeling, it is not clear whether these are neurons or glia.

Cx35/36 is a neuronal connexin—it is enriched in the retina and in neurons and is not found in non-neuronal tissues (O'Brien et al., 1998; Söhl et al., 1998). Cx36 has been shown to preferentially localize in neural–neural gap junctions and is absent in astrocytes and oligodendrocytes (Rash et al., 2001). Surprisingly, here we found Cx35 in radial glia lining the rhombencephalic ventricle beginning at 5 dpf. Though we found cell bodies in the same region at 2 dpf, these could not be unequivocally identified as radial glia at 2 dpf. Nevertheless, Cx35 has been previously shown to be present in cell lines derived from teleostian radial glia (Wen et al., 2010) and in clusters of cells lining the ventricular zone in mice (Lo Turco and Kriegstein, 1991; Bittman et al., 1997). We propose that Cx35-containing gap junctions might be involved in the exchange of signaling factors during proliferation, migration and differentiation of neurons. It has been previously observed that neurons of similar age acquire the same neurotransmitter phenotype and have similar morphological properties (Kinkhabwala et al., 2011). Neuronal lineage has been shown to govern early transient electrical coupling and subsequent excitatory synaptic connectivity in neocortex (Yu et al., 2012). Early Cx35-mediated gap junctional coupling of neural precursors along the ventricular wall might be important for specifying uniform properties across populations of newly-born neurons.

### Conflict of interest statement

The authors declare that the research was conducted in the absence of any commercial or financial relationships that could be construed as a potential conflict of interest.

## Abbreviations

AC: Anterior commissure
CCe: Corpus cerebelli
Chab: Habenular Commissure
Ctec: Tectal commissure
Di: Diencephalon
Hab: Habenula
Hb: Hindbrain
Hy: Hypothalamus
M: Mauthner neuron
ML: Molecular layer
OB: Olfactory bulb
OT: Optic Tectum
P: Pineal
Pd: Dorsal pallium
POC: Post-optic commissure
Pv: Ventral pallium
RG: Radial glia
RV: Rhombencephalic ventricle
SOT: Supra optic tract
TPOC: Tract of the post-optic commissure
TelV: Telencephalic ventricle.
